# Microplastic and the Enteric Nervous System: Effect of PET Microparticles on Selected Neurotransmitters and Cytokines in the Porcine Ileum

**DOI:** 10.3390/ijms252111645

**Published:** 2024-10-30

**Authors:** Ismena Gałęcka, Jarosław Całka

**Affiliations:** 1 Department of Epizootiology, Faculty of Veterinary Medicine, University of Warmia and Mazury in Olsztyn, Oczapowskiego 13, 10-719 Olsztyn, Poland; 2 Department of Clinical Physiology, Faculty of Veterinary Medicine, University of Warmia and Mazury in Olsztyn, Oczapowskiego 13, 10-719 Olsztyn, Poland

**Keywords:** pig, gastrointestinal tract, neurotransmitter, neuropeptide, one health, ingestion, environmental pollution, neuronal plasticity

## Abstract

Microplastic is an environmental hazard to which both animals and humans are exposed. Current reports show that it can cause inflammation, including in the gastrointestinal tract. To examine the impact on the ileum, 15 eight-week-old gilts (five individuals/group) were exposed to PET microplastics (7.6 µm–416.9 µm) at a dose of 0.1 g/day or 1 g/day for 28 days. The collected ileum fragments were investigated for the cytokine concentrations (IL-1β, IL-6, IL-8, IL-10, and TNF-α; ELISA test), neuron populations (cocaine and amphetamine-regulated transcript, galanin, neuronal nitric oxide synthase, substance P, vesicular acetylcholine transporter, and vasoactive intestinal peptide; immunofluorescence staining), and morphometric parameters (histological analysis). Under the influence of MP-PET, there was a reduction in the populations of CART- and GAL-positive neurons in the submucosal plexuses and of nNOS-, VAChT-, and VIP-positive neurons in all the plexuses. In contrast, there was an increase in GAL-positive neurons in the myenteric plexus and SP-positive neurons in all the plexuses. The concentrations of IL-1β, IL-6, IL-8, IL-10, and TNF-α did not undergo statistically significant changes under the influence of the low or high dose of MP-PET. The changes in the histological structure exclusively concerned the thinning of the mucosa and the muscularis externa. The results support the thesis that MP-PET is not neutral to the ileal cells.

## 1. Introduction

The term “microplastics” is no longer unfamiliar to the public. Microplastics are synthetic particles of primary or secondary origin, transparent or colored, regularly or irregularly shaped [1,2]. When defining these particles, the size range is the biggest problem. According to various authors, in terms of size, microplastics fall into the range of 0.1–1000 µm [3] or 0.1–5000 µm [1,2]. The most common definition provides that microplastics should be regarded as particles with a diameter of 5 mm or smaller [2]. In addition, one can also distinguish nanoplastics, mesoplastics, macroplastics, and megaplastics [3,4], the size range of which can also vary depending on the source. For this reason, the authors believe that an authoritative body should establish a single binding definition to facilitate research and the interpretation of results. This would allow misunderstandings to be avoided in further discussions between researchers. When the phrase “microplastic” is entered into the PubMed, Web of Science, and Scopus databases, an increasing trend in the number of scientific publications on this topic can be observed year after year. The scope of research presented in the literature is wide. The publications on the topic include the results of in vivo and in vitro studies concerning the occurrence, characteristics, hazards, degradation, effects on different ecosystems and living organisms, etc. Materials of different origins are used, including particles of a certain size/shape or their mixtures and different concentrations/doses based on the unit weight or the number of particles. Despite the upward trend in the ongoing research, there is still a large cognitive gap. 

The current study focused on one polymer—polyethylene terephthalate (PET). Plastic packaging is widely used in the food industry because of its convenience (high strength, durability, and low weight). It is estimated that until an alternative to PET packaging is invented, the demand for it will continue to increase [5]. Modern lifestyles, i.e., the popularity of dietary (box) catering, ready-to-eat meals, and “fast fashion”, contribute to the environmental release of an increasing number of particles of various sizes, including the above-mentioned polyethylene terephthalate (MP-PET). On the other hand, despite the increased use of plastic products, it should be noted that public awareness of the microplastic problem is growing. As early as 2019, the European Union introduced Directive (EU) 2019/904 of the European Parliament and of the Council of 5 June 2019 on the reduction in the impact of certain plastic products on the environment, and pursuant to it, certain states phased out certain articles, e.g., plastic straws. These measures aim to reduce the amount of particles released into the environment and the volume of plastic waste. It should be noted that plastics, whether on a microscale or a macroscale, can have a harmful effect, but they can also be vectors for heavy metals or antibiotic resistance genes [3]. 

The One Health Initiative aims to take a multidisciplinary approach to health and demonstrates the links between the environment, animals, and humans [6]. In line with this concept, it was decided to use the pig (*Sus scrofa domestica*) as a research model. Pigs can link environmental microplastics and humans through the food chain. Corte Pause et al. noted that livestock [3] could represent the missing link in the exposure of humans to microplastics. The intake of products of animal origin, e.g., meat or milk, can be a source of microplastics for humans [3]. The presence of nanoparticles and/or microparticles (NMP) has also been demonstrated in vegetables and fruit [7], which can be used for animal feeding but can also be directly consumed by humans. For plants, on the other hand, it is the soil that can be a source of contamination [3]. Another source of NMP can be packaging for animal feed and human food [2,8]. In addition, due to its similarity to humans in terms of physiology, anatomy, immunology, or the genome, the pig is used in biomedical research [9]. This allows one to assess the potential effect of NMP on the entire body and draw conclusions about its effects on humans. The above-mentioned methodology for carrying out the research has both advantages and disadvantages as due to the rules restricting the use of animals for scientific research, the size of study groups is limited. In vivo studies using isolated research models, e.g., specific tissues, are more unpredictable than in vitro studies. They are also inconclusive in nature as it is not always possible to explain the changes observed, or they may not be as significant when considering the organism as a single system striving to maintain homeostasis. 

The gastrointestinal tract is constantly exposed to various xenobiotics, and the exposing factors can include food or drink, for example. Xenobiotics and pathophysiological conditions can affect not only the histological structure but also hormone balance, as well as the immune and nervous systems [10]. Xenobiotic-induced changes in one system may result in changes observed in another system, e.g., cytokines may regulate neurotransmission [11], and neurotransmitters/neuropeptides may act as immune modulators [12]. 

The ileum plays an important role in introducing xenobiotics via the alimentary route. The absorption of nutrients and other substances not absorbed in the duodenum and jejunum occurs in the ileum. In the mucosa and submucosa, Peyer’s patches are found, which are one of the components of the gut-associated lymphoid tissue (GALT) [13,14], which can account for up to 70% of the mass of the immune system [14]. Peyer’s patches contain M cells capable of collecting an antigen from the intestinal lumen and presenting it to the T and B cells via the dendritic cells. This way, NMP particles can be presented to the immune system cells. Therefore, due to its function, the ileum can also be exposed to MP-PET. In order to compare the effect of MP-PET on cytokine concentration, those that were tested in other experimental systems were selected [4]. Mainly pro-inflammatory cytokines (IL-1β, IL-8, IL-6, and TNF-α) and IL-10, as a representative of anti-inflammatory cytokines, were selected.

Based on previous studies by the authors, it is known that MP-PET affects the enteric nervous system (ENS) [15,16], which is also referred to as the second brain [17,18]. The term “second brain” stems from the fact that ENS is a component of the autonomic nervous system—a network of neurons and glial cells in the gastrointestinal tract that regulate most functions [17,18]. The ENS comprises a myenteric plexus that mainly regulates the contractile and relaxation functions and the submucous plexus, which affects the regulation of blood flow or the secretory functions [17]. It should be remembered, however, that this is a great oversimplification of the functions of the myenteric and submucous plexus. In pigs and humans, the submucous plexus is divided into the inner (ISP) and the outer (OSP) submucous plexus [19]. Cellular damage in the ENS and its abnormal function can be congenital, e.g., in the course of Hirschsprung’s disease [17,18], or secondary, e.g., caused by gastroparesis [17] or inflammation [20]. The results obtained to date [15,16] show that despite using tissues derived from the same animals, the plasticity of the enteric nervous system neurons varies depending on the gastrointestinal tract segment under study. Therefore, the authors decided to carry out a similar study on the ileum, which would provide a comprehensive overview of the effects of microplastics on the small intestine. The selection of cocaine and amphetamine-regulated transcript (CART), galanin (GAL), neuronal nitric oxide synthase (nNOS; nitrergic neuron marker), substance P (SP), vesicular acetylcholine transporter (VAChT; cholinergic neuron marker), and vasoactive intestinal peptide (VIP) was related to the functions performed by these neurotransmitters and neuropeptides in both pathological and physiological conditions. They not only regulate blood flow, secretory functions, and intestinal motility but may also have neuroprotective effects or modulate the immune response [19,20].

In vivo and in vitro studies conducted to date confirm that NMP can exhibit pro-inflammatory effects [4,21], e.g., by inducing oxidative stress [22,23,24] or cytotoxicity [6]. The effect of NMP may vary depending on the size and the material it is made from [4]. Thus, the authors chose to use particles representing a heterogeneous mixture in the study, even though they were composed of a single material. This was an advantage of this study as it more closely simulated the actual hazard than a study using exclusively one particle size. 

This study aimed to determine if and how 28-day exposure to microplastics at doses of 0.1 and 1 g/animal/day administered orally affected the population size of the CART-, GAL-, nNOS-, SP-, VAChT-, and VIP-positive neurons; the concentrations of IL-1β, IL-6, IL-8, IL-10, and TNF-α; and the histological structure of the pig ileum.

## 2. Results

### 2.1. Cytokine Concentrations

**Figure 1:**
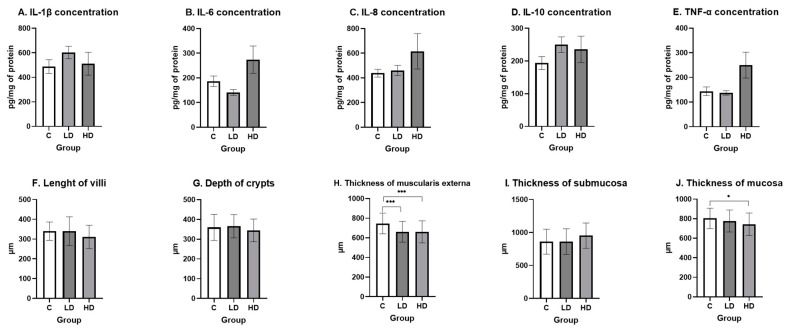
Concentrations of selected cytokines in the ileal wall (**A**–**E**) and the morphometric indices (**F**–**J**) of the ileal wall. The cytokine concentration results are presented as a mean (pg/mg protein) ± SEM. The morphometric measurement results are presented as a mean (µm) ± SD. C—control group; LD—low-dose group; HD—high-dose group. * *p* < 0.05, *** *p* < 0.001 indicate statistically significant differences.

The concentrations of the cytokines under study are presented in Figure 1A–E. Although differences in the concentrations of the cytokines under study were observed between the groups, statistical analysis did not show them to be significant (*p* > 0.05). In the LD group, the highest concentration was noted for IL-1β (602.83 pg/mg protein ± 51.12) and IL-10 (250.17 pg/mg protein ± 23.73), whereas the lowest was in IL-6 (141.39 pg/mg protein ± 11.90) and TNFα (137.31 pg/mg protein ± 9.12). On the other hand, the high dose increased the concentration of each cytokine in relation to the study group (IL-1β from 487.95 pg/mg protein ± 56.51 to 511.02 pg/mg protein ± 92.93, IL-6 from 186.59 pg/mg protein ± 21.33 to 273.93 pg/mg protein ± 55.50, IL-8 from 438.18 pg/mg protein ± 31.44 to 615.34 pg/mg protein ± 143.42, IL-10 from 193.75 pg/mg protein ± 19.88 to 253.57 pg/mg protein ± 40.49, and TNFα from 143.77 pg/mg protein ± 17.59 to 249.99 pg/mg protein ± 52.46).

### 2.2. Histological Study

Under the influence of the high dose of MP-PET, there was a thinning of the thickness of the mucosa (from 803.63 µm ± 104.70 to 743.39 µm ± 114.73; *p* < 0.05) and the thickness of muscularis externa (from 746.36 µm ±106.04 to 660.30 µm ±112.69; *p* < 0.001). The thickness of the muscularis externa (661.73 µm ± 105.25; *p* < 0.001) was also lower, as compared with the control group, in animals from the low-dose MP-PET group. As for the other indices, i.e., the length of the villi, the crypt depth, and the thickness of the submucosa, no statistically significant differences were noted (*p* > 0.05). The length of the intestinal villi ranged from 310.66 µm ± 59.00 to 340.10 µm ± 46.05, the crypt depth from 344.37 µm ± 57.32 to 365.70 µm ± 59.13, and the thickness of the submucosa from 860.01 µm ± 188.99 to 953.56 µm ± 191.43 (Figure 1F–J).

### 2.3. Immunofluorescence Staining

#### 2.3.1. Cocaine and Amphetamine Regulated Transcript

**Figure 2:**
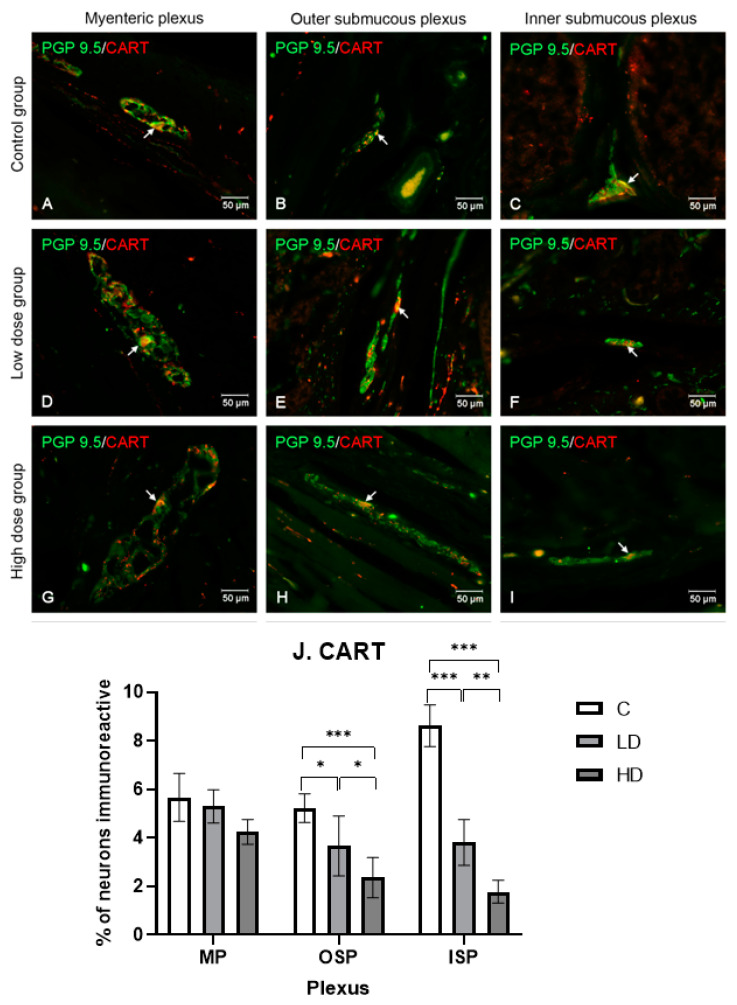
Distribution pattern for neurons immunoreactive toward protein gene product 9.5 and CART. (**A**,**D**,**G**) Myenteric plexus, (**B**,**E**,**H**) outer submucous plexus, and (**C**,**F**,**I**) inner submucous plexus in the C group (**A**–**C**), LD (**D**–**F**), and HD (**G**–**I**). The photographs were created by overlaying two colors (green for PGP 9.5 and red for CART). The arrows indicate cells immunoreactive toward the test substance. (**J**) The percentage of neurons immunoreactive toward CART, expressed as a mean (%) ± SEM. C—control group; LD—low-dose group; HD—high-dose group. * *p* < 0.05, ** p < 0.01, *** *p* < 0.001 indicate statistically significant differences.

CART-positive neurons were among the least numerous populations of the neurotransmitters under study. The myenteric plexus neurons were the only neurons unaffected by the low or high dose of MP-PET. Their percentage ranged between 5.67% ± 0.99 (C) and 4.25% ± 0.51 (HD). However, both doses reduced the CART-positive neuron population size (C 5.23% ± 0.59), with the low dose doing so to a lesser extent (3.67% ± 1.23; *p* < 0.05) than the high dose (2.36% ± 0.83; *p* < 0.001) in OSP. In ISP, however, the reduction in the population size of neurons (C 8.62% ± 0.85) was equally significant under the influence of both MP-PET doses (LD: 3.81% ± 0.94, and HD: 1.78% ± 0.47; *p* < 0.001). The results and microscopic images are presented in the Figure 2.

#### 2.3.2. Galanin

**Figure 3:**
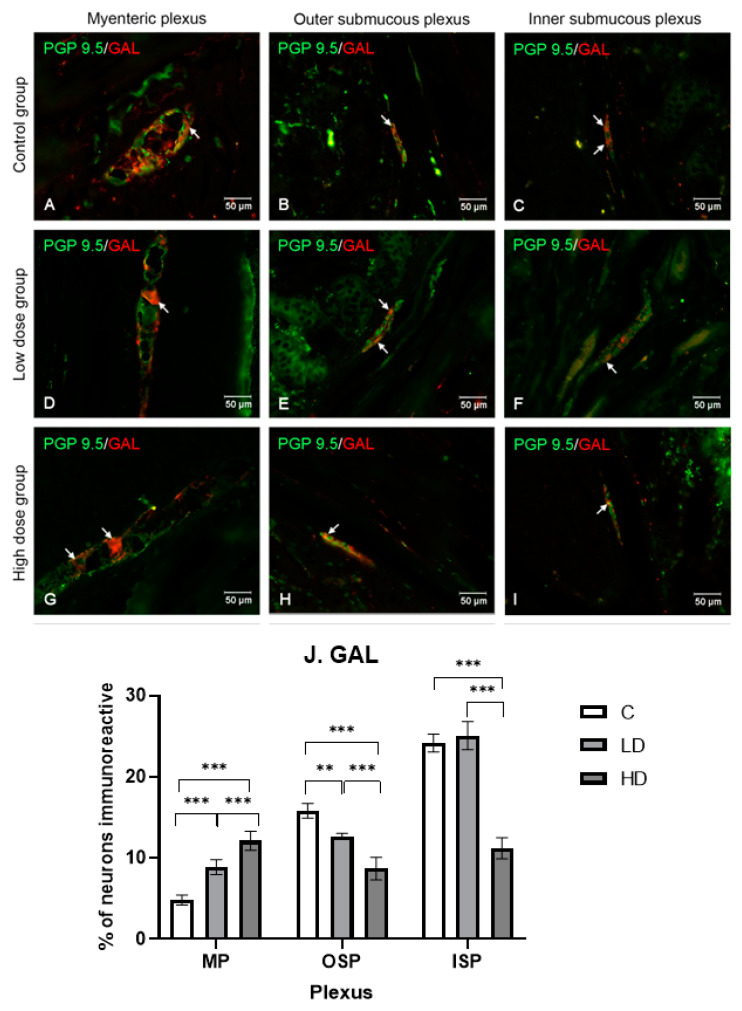
Distribution pattern for neurons immunoreactive toward protein gene product 9.5 and GAL. (**A**,**D**,**G**) Myenteric plexus, (**B**,**E**,**H**) outer submucous plexus, and (**C**,**F**,**I**) inner submucous plexus in the C group (**A**–**C**), LD (**D**–**F**), and HD (**G**–**I**). The photographs were created by overlaying two colors (green for PGP 9.5 and red for GAL). The arrows indicate cells immunoreactive toward the test substance. (**J**) The percentage of neurons immunoreactive toward GAL, expressed as a mean (%) ± SEM. C—control group; LD—low-dose group; HD—high-dose group ** *p* < 0.01, *** *p* < 0.001 indicate statistically significant differences.

GAL-positive neurons in control animals accounted for 4.86% ± 0.61 (MP), 15.84% ± 0.90 (OSP), and 24.18% ± 1.11 (ISP). Under the influence of the high dose, statistically significant differences (*p* < 0.001) were noted in each plexus under study, namely, in MP (12.13% ± 1.18), in OSP (8.68% ± 1.40), and in ISP (11.19% ± 1.31). The low dose in MP contributed to an increase in the GAL-positive neuron percentage to 8.86% ± 0.92 (*p* < 0.001) and in OSP to a decrease to 12.66% ± 0.37 (*p* < 0.01). The lack of differences was demonstrated in ISP (25.10% ± 1.73, *p* > 0.05). The results and microscopic images are presented in the Figure 3. 

#### 2.3.3. Neuronal Nitric Oxide Synthase

**Figure 4:**
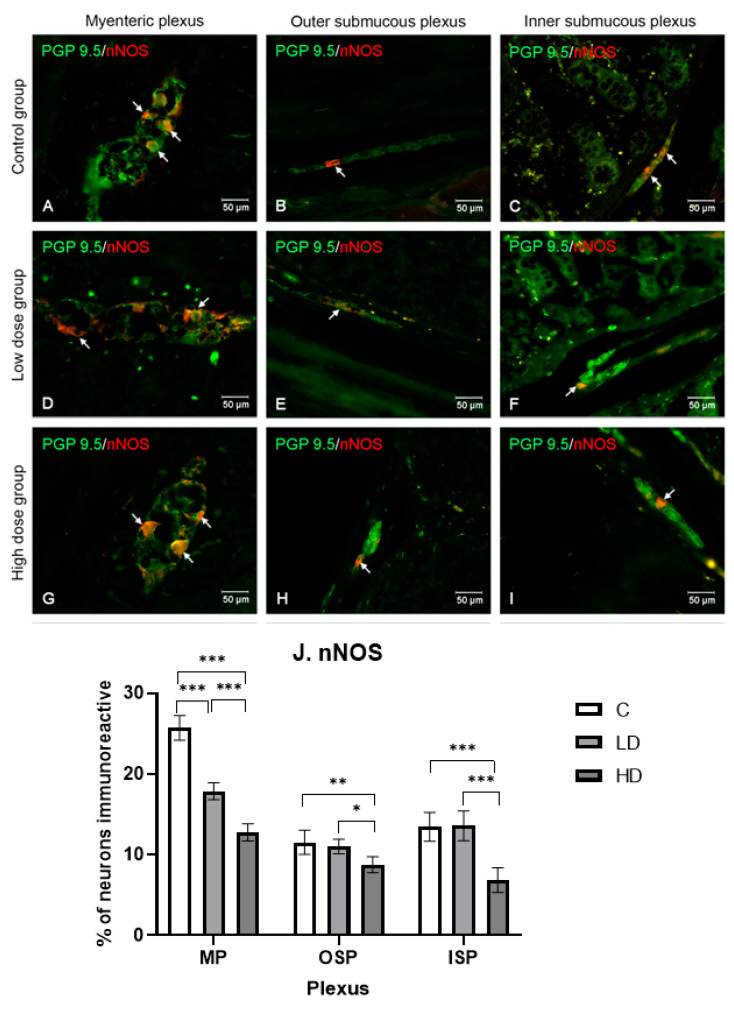
Distribution pattern for neurons immunoreactive toward protein gene product 9.5 and nNOS. (**A**,**D**,**G**) Myenteric plexus, (**B**,**E**,**H**) outer submucous plexus, and (**C**,**F**,**I**) inner submucous plexus in the C group (**A**–**C**), LD (**D**–**F**), and HD (**G**–**I**). The photographs were created by overlaying two colors (green for PGP 9.5 and red for nNOS). The arrows indicate cells immunoreactive toward the test substance. (**J**) The percentage of neurons immunoreactive toward nNOS, expressed as a mean (%) ± SEM. C—control group; LD—low-dose group; HD—high-dose group. * *p* < 0.05, ** *p* < 0.01, *** *p* < 0.001 indicate statistically significant differences.

In the control group, the percentage of nNOS-positive neurons accounted for 25.74% ± 1.53 (MP), 11.54% ± 1.51 (LD), and 13.47% ± 1.79 (HD). The high dose of MP-PET contributed to a decrease in the nNOS-positive neuron population in all the plexuses, in MP to 12.78% ± 1.06 (*p* < 0.001), in OSP to 8.75% ± 1.00 (*p* < 0.001), and in ISP to 6.87% ± 1.52 (*p* < 0.001). However, the low dose of MP-PET only resulted in a difference in MP, 17.90% ± 1.05 (*p* < 0001). In OSP (11.03% ± 0.88) and ISP (13.59% ± 1.86), no statistically significant differences (*p* > 0.05) under the influence of LD MP-PET were noted. The results and microscopic images are presented in the Figure 4. 

#### 2.3.4. Substance P

**Figure 5:**
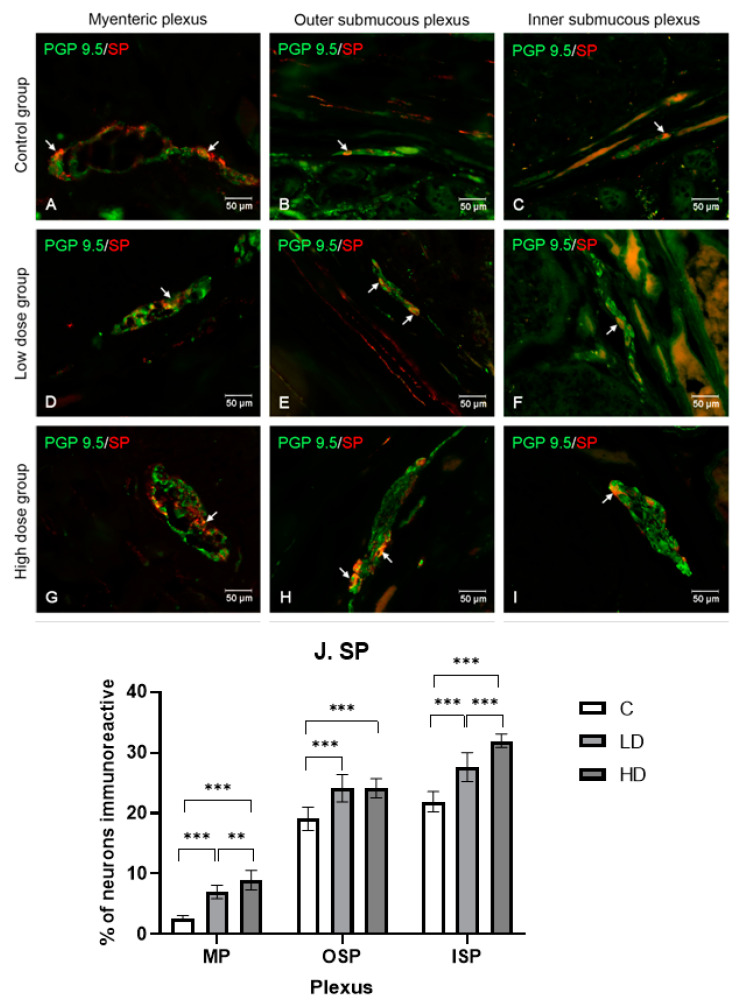
Distribution pattern for neurons immunoreactive toward protein gene product 9.5 and SP. (**A**,**D**,**G**) Myenteric plexus, (**B**,**E**,**H**) outer submucous plexus, and (**C**,**F**,**I**) inner submucous plexus in the C group (**A**–**C**), LD (**D**–**F**), and HD (**G**–**I**). The photographs were created by overlaying two colors (green for PGP 9.5 and red for SP). The arrows indicate cells immunoreactive toward the test substance. (**J**) The percentage of neurons immunoreactive toward SP, expressed as a mean (%) ± SEM. C—control group; LD—low-dose group; HD—high-dose group. ** *p* < 0.01, *** *p* < 0.001 indicate statistically significant differences.

Under the influence of the low and high doses of MP-PET, the percentage of SP-positive neurons increased in all the plexuses (*p* < 0.001). In the MP, in the C group, the percentage was 2.68% ± 0.39, 6.99% ± 1.12 in the LD group, and 8.94% ± 1.62 in the HD group. In OSP, in the C group, 19.07% ± 1.91 SP-positive neurons were noted; in OSP, 24.79% ± 2.24; and in ISP, 24.13% ± 1.59. Similarly, changes occurred in ISP, i.e., in the C group, 21.90% ± 1.68; in OSP, 27.61% ± 2.41; and in ISP, 31.96% ± 1.11. The results and microscopic images are presented in the Figure 5. 

#### 2.3.5. Vesicular Acetylcholine Transporter 

**Figure 6:**
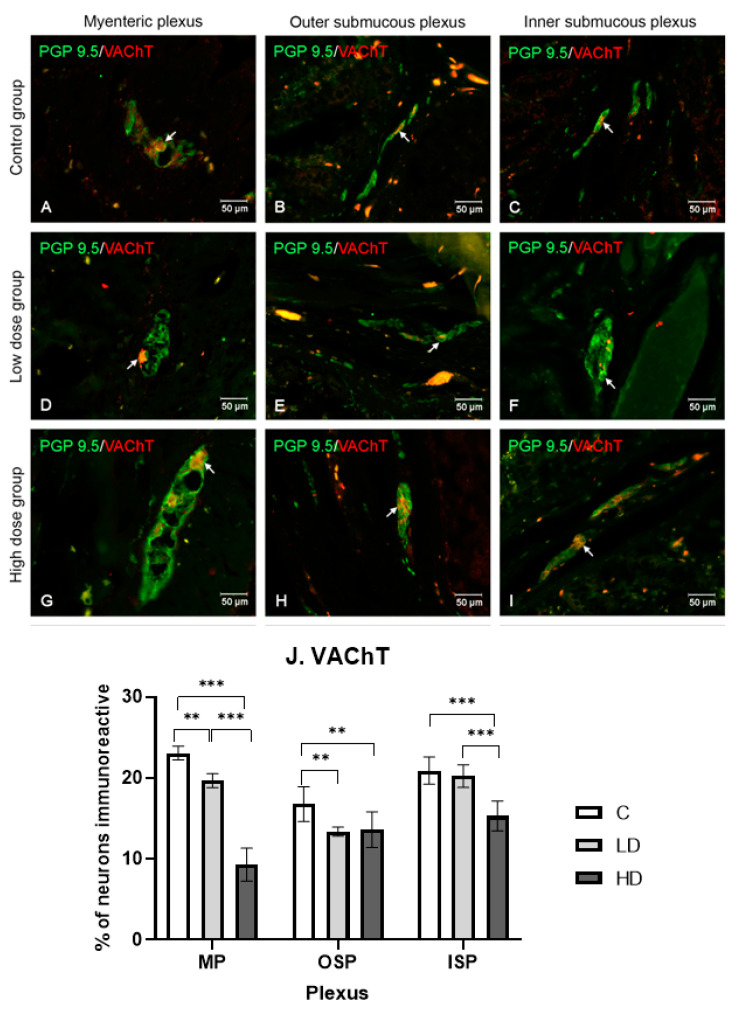
Distribution pattern for neurons immunoreactive toward protein gene product 9.5 and VAChT. (**A**,**D**,**G**) Myenteric plexus, (**B**,**E**,**H**) outer submucous plexus, and (**C**,**F**,**I**) inner submucous plexus in the C group (**A**–**C**), LD (**D**–**F**), and HD (**G**–**I**). The photographs were created by overlaying two colors (green for PGP 9.5 and red for VAChT). The arrows indicate cells immunoreactive toward the test substance. (**J**) The percentage of neurons immunoreactive toward VAChT, expressed as a mean (%) ± SEM. C—control group; LD—low-dose group; HD—high-dose group. ** *p* < 0.01, *** *p* < 0.001 indicate statistically significant differences.

The VAChT-positive neurons were the most abundant in the MP (23.09% ± 0.84 (the C group)), followed by ISP (20.92% ± 1.68 (the C group)). They were the least abundant in OSP (16.77% ± 2.14 (the C group)). The low dose of MP-PET reduced the positive neuron percentage to 19.67% ± 0.87 in MP and to 13.38% ± 0.56 in OSP (*p* < 0.01). No statistically significant changes were noted in ISP (20.32% ± 1.40, *p* > 0.05). Under the influence of the high dose of MP-PET, the greatest differences were observed in MP (9.30% ± 2.04) and ISP (15.32% ± 1.85) (*p* < 0.001). In the OSP, the differences were less pronounced than the previously mentioned plexuses (13.38% ± 0.56) (*p* < 0.01). The results and microscopic images are presented in the Figure 6.

#### 2.3.6. Vasoactive Intestinal Peptide 

**Figure 7:**
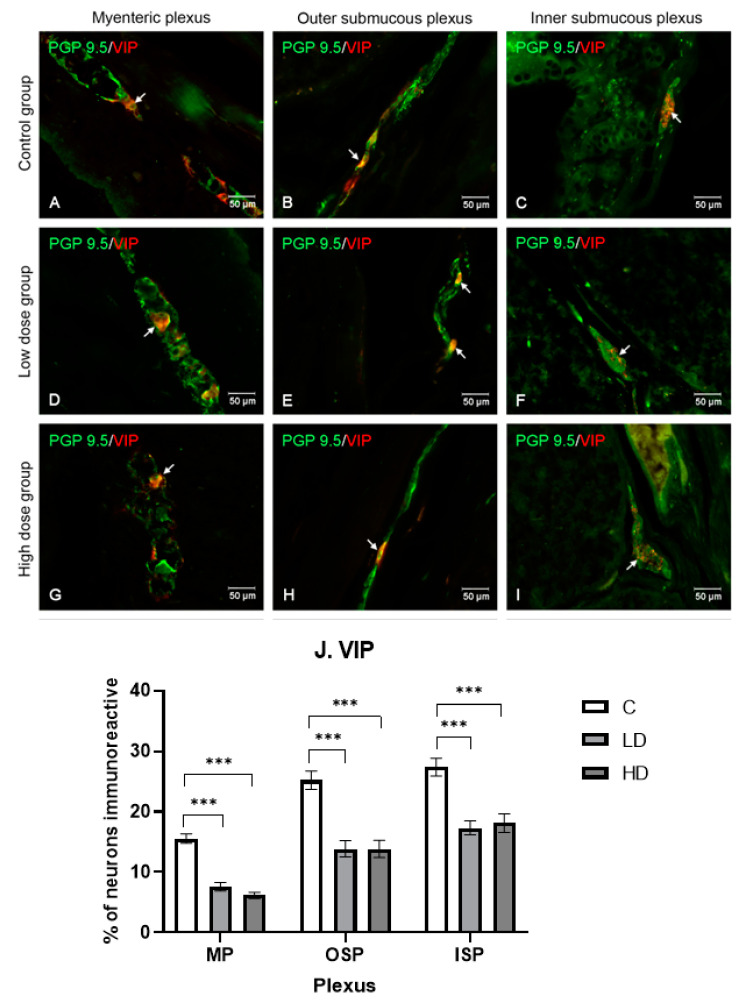
Distribution pattern for neurons immunoreactive toward protein gene product 9.5 and VIP. (**A**,**D**,**G**) Myenteric plexus, (**B**,**E**,**H**) outer submucous plexus, and (**C**,**F**,**I**) inner submucous plexus in the C group (**A**–**C**), LD (**D**–**F**), and HD (**G**–**I**). The photographs were created by overlaying two colors (green for PGP 9.5 and red for VIP). The arrows indicate cells immunoreactive toward the test substance. (**J**) The percentage of neurons immunoreactive toward VIP, expressed as a mean (%) ± SEM. C—control group; LD—low-dose group; HD—high-dose group. *** *p* < 0.001 indicate statistically significant differences.

In the control group, 15.57% ± 0.78 (MP), 25.21% ± 1.53 (LD), and 27.38% ± 1.47 VIP-positive neurons were noted. Both the low and the high doses of MP-PET resulted in statistically significant differences at *p* < 0.001 in all plexuses. In MP, there was a reduction in the neurons immunoreactive toward VIP, to 7.59% ± 0.71 (LD) and 6.17% ± 0.51 (HD). In ISP, however, there was a reduction to 17.33% ± 1.16 (LD) and 18.10% ± 1.54 (HD), and in OSP, there was a reduction to 13.86% ±1.34 (LD) and 13.85% ±1.42 (HD). The results and microscopic images are presented in the Figure 7. 

## 3. Discussion

Reports on the effects of NMP on pigs are currently very scarce. They mainly focus on the identification of NMP in feces [25] or tissues [26] or the use of porcine tissues in in vitro models [27,28,29]. In contrast to the fairly well-understood effect on mice, rats, or aquatic animals, the effect on pigs still appears enigmatic. In addition, only recently have more studies reported using PET in research. For these reasons, it is difficult to determine the exact mechanism behind the occurrence of the observed changes. Most studies on the effect of NMP report changes in the intestinal barrier integrity [23] and an increase in pro-inflammatory cytokines [4,23] or an increase in the oxidative stress marker levels [4,23,28,29]. They also report higher toxicities for smaller particles [30]. In addition to the authors’ articles, only single studies using pigs as the in vivo research model are available. However, they use different types of plastics, animal ages, and doses. Studies using polystyrene microplastics have shown that they contribute to diarrhea, impaired angiogenesis in the gut, and impaired meat quality [31,32]. The difference concerned the age of the animals used, the dose, and the particle size. According to the research, the piglets received an average of approximately 632.25 mg and 1198.5 mg of microplastics when reaching their final body weight. The current study used a daily dose rather than a dose per kg body weight since the authors believed this more closely represented environmental exposure [33]. Since compared with the above-mentioned studies, the daily dose may appear to be a much milder dose (the low dose is lower by approximately 250 times, and the high dose is lower by approximately 50 times), it is possible not to observe such significant changes as those observed in the above-mentioned studies [31,32]. However, according to the results, even such low doses can induce changes in the ENS.

Given the reported pro-inflammatory effect of NMP, it might be expected that the percentage of GAL-, VIP-, nNOS-, SP-, or CART-positive neurons would be increased after oral exposure, which is linked to the neuroprotective properties of these neurotransmitters and neuropeptides [34,35,36,37]. The available results, however, show that the population of these neurons decreased, except for the GAL-positive neurons in MP and SP-positive neurons in all plexuses. Similarly, one could suspect that the pro-inflammatory cytokine levels should increase. Other studies indicate that NMP can increase pro-inflammatory cytokine levels [23] but may not induce any statistically significant changes [4,38]. It should be noted, however, that PET was not used in any of the cases cited. The results obtained by the authors show certain upward trends: it can be observed that with an increase in the MP-PET dose, the concentrations of TNFα, IL-6, and IL-8 increase, with the *p* value, however, remaining at a level of >0.05. 

It can also be assumed that MP-PET does not affect all the neurotransmitters and neuropeptides in the same way. The lack of differences for CART in MP and nNOS between the C group and the LD group in OSP and ISP may either be indicative of this fact or provide evidence of changes that are only just developing, as is the case of cytokine concentrations under the influence of the mycotoxin zearalenone, where differences in cytokine levels are shown in the ileum of pigs of similar body weight [39]. Analysis of the results shows that the cytokine levels may vary depending on the day of testing. When comparing the IL-1β concentration on days 14, 28, and 42, one can observe that on day 28, there was a decrease in the concentration, as compared with day 14, and on day 42, an increase took place again [39]. The same was true for IL-8 or TNFα [39]. Further research is needed to confirm whether the dose of MP-PET chosen by the authors would contribute to significant changes but under longer exposure or whether the doses chosen are incapable of producing a noticeable effect. Given the differences observed in the percentage of neurons, the authors believe that the 4-week exposure may have contributed to neuroplasticity changes but without significant immune system stimulation. Alternatively, it is also possible that the 28-day exposure contributed to a faster adaptation of the immune system and a return to homeostasis than ENS and hence the observed discrepancies between the studied plexuses or other sections of the small intestine. 

Microplastics have been shown to contribute to changes in the expression and activity of such neurotransmitters as dopamine, glutamate, serotonin, gamma-aminobutyric acid, and acetylcholine [21,40]. The neurotoxicity of NMP has been linked to the effect on neurotransmission, e.g., by affecting the expression of the genes involved [24,40] or reducing nerve cells [41]. MP-PET appears to affect VAChT-, nNOS-, and VIP-positive neurons equally, irrespective of the small intestine segment. The differences presented in this study not only are due to the potential changes in the physical and/or chemical structure of microplastic but also may confirm that the function of neurotransmitters is determined not only by the segment of the gastrointestinal tract but also by the plexus. 

The level of expression of neurotransmitters and neuropeptides can also be affected by time and the tissue fragment examined, even of the same gastrointestinal tract segment. Studies showing the colocalization of CART- and/or GAL-positive neurons in a stomach with adenocarcinoma have demonstrated that the number of these fibers increased in the neoplastic area compared with the area showing no abnormal cells [42]. On the other hand, a study on the seabream (*Sparus aurata*) brains observed that changes in the levels of brain monoaminergic neurotransmitters after a 30-day period of microplastic detoxification showed a tendency to normalization [43]. Considering that the likely exposure to NMP in daily life is not the same every day, this provides an opportunity for ENS to adapt smoothly to this xenobiotic.

A study comparing PET and polyethylene showed that PET may exhibit potentially greater toxic properties, e.g., due to its superior oxidative properties and its capability of dissociating guanine–cytosine nucleoside pairs, which may affect DNA replication [44]. Additionally, nanoplastics were demonstrated to exhibit the capability of affecting miRNA [45]. The differences in the percentage of neurons positive to the test substances may be due to abnormal expression of miRNA, for example, which may regulate the cell cycle, growth, development, or apoptosis of cells in ENS [46]. Polystyrene microplastics are known to disrupt intestinal angiogenesis, specifically by destabilizing the mRNA of angiogenic factors [31]. In addition, it was also demonstrated that nanoparticles can contribute to abnormalities in the protein folding process [47] or damage the synapses [48]. Abnormalities may also concern neurotransmitter receptors [49]. Polystyrene nanoplastics have been noted to exhibit different toxicities depending on the cells/tissues under study [22]. Changes in the plasticity of enteric nervous system neurons and neurotransmission [50], changes in protein folding [47], and changes in the gut microflora [51] may be responsible for the development of neurodegenerative diseases. NMP contaminants could potentially be a factor that promotes the development of neurodegenerative or neurodevelopmental diseases [48], but this requires further research. Under the influence of digestion, the microplastic’s physicochemical properties may change, which could be the reason for the observed differences in different segments of the small intestine or the entire gastrointestinal tract.

Changes in the thickness of the muscularis externa and the population of VAChT-, VIP-, and CART-positive neurons may indicate changes occurring in the motor activity of the ileum under the influence of MP-PET. Interestingly, the thinning and the smaller population of VAChT-positive neurons may indicate reduced contractility as acetylcholine is one of the main factors stimulating smooth muscle contraction, similar to SP [52]. When exposed to polystyrene, acetylcholine production in mice is reduced [53]. On the other hand, VIP and NO are the main factors causing the relaxation of the muscle layer [52], and these are reduced under the influence of MP-PET. Therefore, this should result in increased intestinal contractility, especially with an increase in the percentage of SP-positive neurons. However, other mechanisms involved are not the subjects of the study presented here. During the experiment, no clinical changes in motility, manifested as constipation or diarrhea, were noted, but smooth muscle contractility tests would be required to confirm changes in intestinal motility. In addition, there are reports that CART can promote the survival of neurons of the muscularis mucosae and/or smooth muscles [54]. In the course of Hirschsprung disease, the population size of CART-positive neurons decreases [55], similar to the present study, where the same was noted in the submucosal plexus area. Interestingly, a downward trend in the CART-positive neuron population size was noted in the MP, but this was not statistically significant (*p* > 0.05). 

Wang et al. studied the effects of low-density polyethylene microplastics, or their oxidized form, on the microbiome–gut–brain axis. They demonstrated that acetylcholine levels were significantly reduced in the cerebral cortex and the hippocampus, and acetylcholinesterase activity was significantly increased, indicating abnormalities in neurotransmission and that a similar mechanism underlies Alzheimer’s disease [50]. An additional aspect that makes the microplastic-induced changes similar to neurodegenerative diseases, in addition to changes in the neurotransmitter levels, includes changes in the histological picture, changes in the intestinal barrier integrity, or the stimulation of the immune system [21]. The presented study also suggests that microplastics may be a factor that will promote the occurrence of neurodegenerative diseases, e.g., Alzheimer’s disease.

If further research is conducted into the effects of NMP on neurons and neuronally active substances, and if results similar to those presented in this article are demonstrated, the use of endogenous VIP, for example, may be part of counteracting the negative effects of exposure to NMP. This is all the more important because VIP can affect the intestines and the entire body [56]. 

Limitations of the presented research concern the small size of the study group, which, however, is related to the reduction in the use of animals in in vivo research. In addition, the research model is based on the use of animals of the same breed originating from the same farm. It was shown that the origin and breed of animals can influence the level of neurotransmitters [57] and the gut microbiome [58]. The study results presented here only show the potential effect of MP-PET without investigating the mechanism behind them. Additionally, in the analysis of the results, *p* values were prioritized as a measure of statistical significance. Due to the exploratory nature of the studies and limitations related to sample sizes (*n* = 5), the effect magnitudes of the tests used may be potentially compromised.

The results support the thesis that MP-PET is not neutral to the ileal cells. Even such low doses are capable of inducing an ENS response in the form of changes in the population of neurons immunoreactive toward CART, GAL, nNOS, SP, VAChT, and VIP. Under the influence of MP-PET, there is a reduction in the populations of CART- and GAL-positive neurons in the submucosal plexuses and of nNOS-, VAChT-, and VIP-positive neurons in all the plexuses. In contrast, there is an increase in GAL-positive neurons in the myenteric plexus and SP-positive neurons in all the plexuses. The concentrations of IL-1β, IL-6, IL-8, IL-10, and TNF-α did not undergo statistically significant changes under the influence of the low or high dose of MP-PET. However, the changes in the histological structure exclusively concerned the thinning of the mucosa and the muscularis externa. Undoubtedly, further research focusing on the effects of NMP in the gastrointestinal tract should be carried out as it may contribute to building a strategy for coping with NMP in humans and animals. 

## 4. Materials and Methods

The procedure for carrying out the experiment, performing double immunofluorescence staining, and conducting histological examinations was followed according to the protocol and using the reagents indicated in publications by Gałęcka et al. [15,16]. 

### 4.1. Microplastic

The characterization of PET (Cat. No ES306031/1, Goodfellow Cambridge Ltd., Cambridge, UK) in powder form was carried out using a Mastersizer 2000 analyzer (Malvern Instruments Ltd., Worcestershire, UK) and a Phenom ProX G6 scanning microscope (ThermoFisher Scientific, Waltham, MA, USA). This analysis found that 10% of the particles under study had a diameter of less than 51.6 µm, 50% had a diameter of less than 124.6 µm, and 90% had a diameter of less than 237.0 µm. The test sample contained particles with diameters ranging from 7.6 µm to 416.9 µm, with the predominant proportion of particles with a diameter of 158.5 µm. Analysis of the microscopic image showed that the used powder was a mixture of particles of different surface areas, sizes, and shapes. The characteristics of the used particles are provided in previous publications [16].

Two doses were applied: a low dose (LD) of 0.1 g/day and a high dose (HD) of 1 g/day. They were administered daily in gelatine capsules before the morning feeding. The LD dose represented a realistic exposure to microplastics (according to Senathirajah et al., 0.1–5 g microplastics/week/person [33], translating into 0.01–0.71 g microplastics/day/person). In addition, when establishing the low dose, the study by Deng et al. was used as a guide [59]. Based on this study, the dose was adjusted to the weight of the pigs (approximately 20 kg b.w.). The HD dose, on the other hand, was calculated based on the decimal algorithm (for log(0,1)  =   −1; for log(1)  =  0) and was a dose ten times higher than the LD dose. 

### 4.2. Animals

All procedures were based on the approval given by the Local Ethical Commission (decision no. 10/2020 of 26 February 2020) and in accordance with the Polish law, which sets out the conditions and methods of conducting animal experiments in connection with the Act of 15 January 2015 on the Protection of Animals for Scientific or Educational Purposes of (Journal of Laws 2015, No 266), applicable in the Republic of Poland, and Directive 2010/63/EU of the European Parliament and of the Council of 22 September 2010 on the Protection of Animals Used for Scientific Purposes. 

The experiment used 15 sexually immature gilts (*Sus scrofa domestica*) of the Pietrain X Duroc breed, from a farm in Lubawa (Poland), aged approximately 8 weeks, which were divided into three study groups (*n* = 5). The assignment of animals to research groups was performed using a computer random order generator. The first group (C) was administered empty gelatine capsules, the second group received the low microplastic dose (LD), and the third group received the high microplastic dose (HD). Animal handling started with animals from the control group, then LD and HD, and the person taking care of the animals was not informed about the animals’ affiliation to the research group. After a one-week acclimatization period, the pigs were subjected to a 28-day oral exposure to microplastics. Throughout the period, they were kept in three pens containing five animals each, with no contact between individuals from different groups. Room temperature was maintained at 21 ± 1 °C and relative humidity at 57.5 ± 2.5%. A 12 h light/dark cycle was used. Feed was administered twice a day and water ad libitum. Stainless steel feeders were used for animal feeding and watering. The animals were kept in the premises of the Faculty of Veterinary Medicine of the University of Warmia and Mazury in Olsztyn

After 28 days, all the animals were euthanized. The animals were administered intramuscularly with atropine (0.05 mg/kg i.m., Polfa, Warsaw, Poland), xylazine (3 mg/kg, i.m., Vet-Agro, Lublin, Poland), and ketamine (6 mg/kg, i.m., Vetoquinol Bio-wet, Gorzów Wlkp., Poland) [60]. When their unconsciousness was confirmed, their vital functions were arrested by an overdose of sodium pentobarbital (0.6 mL/kg i.v., Biowet, Puławy, Poland). Tissue collection started once the absence of corneal reflex, pulse, and respiratory and cardiac murmurs was confirmed. Segments of the ilium, located 2 cm upstream of the ileocecal valve, were collected for further testing.

### 4.3. Determination of Cytokine Concentrations

Immediately after the collection, the intestinal segments were frozen in liquid nitrogen and kept at −80 °C until the performance of analyses. Each sample (1 g) was homogenized using an extraction buffer consisting of PBS (Cat. No. P4417, Sigma-Aldrich, Saint Louis, MO, USA), protease inhibitors (cOmplete ULTRA Tablets, Mini, EASYpack Protease Inhibitor Cocktail, Cat. No. 05892970001, Sigma-Aldrich, St. Louis, MO, USA), and a homogenizer (Homogenizer T10 Basic Ultra-Turrax IKA Poland Sp. z o.o., Warsaw, Poland). It was then centrifuged at 5000× *g* for 60 min (Eppendorf 5804R centrifuge, Eppendorf Netherlands B.V., Nijmegen, Netherlands), and the resulting supernatant provided the materials for further analysis using commercial ELISA kits (Table 1) in accordance with the manufacturer’s instructions. Previously, the protein concentration was determined using Qubit Protein Assay (Cat. No. Q33211, ThermoFisher Scientific, Waltham, MA, USA) and Qubit 2.0 Fluorometer (Cat. No. Q32866, ThermoFisher Scientific, Waltham, MA, USA) in accordance with the manufacturer’s instructions. Absorbance measurements were taken using a Multiskan FC Microplate Photometer (ThermoFisher Scientific, Waltham, MA, USA) in two replications. The cytokine concentration in the homogenate samples was compared with the protein content.

### 4.4. Histological Study

The tissue samples were fixed in 4% paraformaldehyde solution in 0.1 M phosphate buffer (pH 7.4) for 48 h, dehydrated in ethanol (TP 1020, Leica, Wetzlar, Germany), and embedded in paraffin (EG1150, Leica, Wetzlar, Germany). The 4 µm thick sections were prepared (HM 340E, Microm, Wetzlar, Germany) and stained by the hematoxylin and eosin method (HE) using a multistainer (ST5020+CV5030, Leica, Wetzlar, Germany). The digitization was performed using a Pannoramic 250 Flash scanner (3DHistech, Budapest, Hungary). SlideViewer 2.6 software (3DHistech, Budapest, Hungary) was used for the morphometric analysis (the length of the villi, the crypt depth, the thickness of the mucosa, the thickness of the submucosa, and the thickness of the muscularis externa). The measurements were performed on three sections per animal, separated from each other by at least 1 cm, in 10 replicates per slide, and the mean values were statistically analyzed.

### 4.5. Immunofluorescence Staining

**Figure 8:**
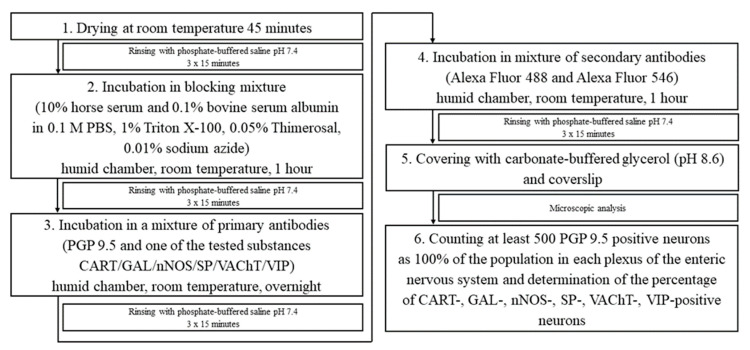
Immunofluorescence staining steps.

The segments for immunofluorescence testing were fixed in a 4% paraformaldehyde solution (pH 7.4). After one hour, the tissues were transferred to 0.1 M phosphate buffer (pH 7.4) for three days. The phosphate buffer was replaced every 24 h. The final step of tissue fixation involved their transfer to an 18% sucrose solution (pH 7.4) for 14 days at 4 °C. The intestinal segments were embedded in OCT Tissue-Tek (Sakura Finerek USA, Inc., Torrance, CA, USA) and, using a cryostat (CM 1860, Leica, Nussloch, Germany), cut at a temperature of −22 °C into 14 µm sections and attached to chrome-alum-coated slides. The staining steps are shown in Figure 8. To visualize the neurons in the ileum, protein gene product 9.5 (PGP 9.5) was used as a pan-neuronal marker. A list of the used antibodies is provided in Table 2. Microscopic analysis was performed using a Zeiss Axio Imager.M2 fluorescence microscope (Zeiss, Oberkochen, Germany), and a camera connected to a PC with ZEISS ZEN 3.0 Microscopy Software (Zeiss, Oberkochen, Germany) was used for image acquisition. To eliminate non-specific labeling, the preabsorption for the neuropeptide antisera with appropriate antigens was performed, and the omission and replacement tests were conducted. The testing performed noted no fluorescence. For the analysis, segments separated from each other by at least 200 µm were used to avoid counting the same cells.

### 4.6. Statistical Analysis

Before statistical analysis, the assumption of linearity and normality was checked. For linearity testing, two-dimensional scatter plots of the analyzed variables were generated. The assumption of normality was confirmed using histograms and residual normality plots. The results are presented as mean ± SEM or mean ± SD. A one-factor analysis of variance (ANOVA) with the Schéffe post hoc test was used to demonstrate the absence or presence of statistically significant differences. The differences were considered significant at a level of *p* < 0.05 (* *p* < 0.05, ** *p* < 0.01, *** *p* < 0.001). The study group was adopted as the independent variable, and the cytokine levels, morphometric indices, and the percentage of neurons immunoreactive toward the test substances were used as the dependent variables. The results were statistically analyzed using Statistica 13.3 software (TIBCO Software Inc., Palo Alto, CA, USA). GraphPad Prism 9.0.0 software (GraphPad Software, Boston, MA, USA) was used for visualization. The graphical abstract was created in BioRender, accessed on 14 October 2024 (BioRender.com/b68d975).

## Figures and Tables

**Table 1:** List of the kits used for ELISA testing.

Antigen	ELISA Kit	Catalog Number	Supplier	Assay Range pg/mL
Tumor necrosis factor α	Porcine TNF-alpha ELISA Kit	orb566582	Biorbyt, Cambridge, United Kingdom	32.25–2000
Interleukin 1β	Porcine IL-1beta ELISA Kit	orb566534		62.5–4000
Interleukin 6	Porcine IL-6 ELISA Kit	orb566538		31.25–2000
Interleukin 8	Porcine IL-8 ELISA Kit	orb566594		15.625–1000
Interleukin 10	Porcine IL-10 ELISA Kit	orb566526		32.25–2000

**Table 2:** List of the antibodies used for immunofluorescence staining.

Primary Antibodies				
Antigen	Species	Working Dilution	Code	Supplier
CART	Rabbit	1:16,000	H-003-61	Phoenix Pharmaceuticals, Inc., Burlingame, CA, USA
GAL	Guinea pig	1:2000	T-5036	Peninsula, San Carlos, CA, USA
nNOS	Rabbit	1:3000	PA1-033	ThermoFisher Scientific, Waltham, MA, USA
PGP 9.5	Mouse	1:1000	480012	
SP	Rat	1:150	8450-0505	BioRad, Hercules, CA, USA
VAChT	Rabbit	1:2000	H-V006	Phoenix Pharmaceuticals, Inc., Burlingame, CA, USA
VIP		1:2000	ab22736	Abcam, Cambridge, United Kingdom
**Secondary Antibodies**				
**Reagents**		**Working Dilution**	**Code**	**Supplier**
Alexa Fluor 488	Donkey anti-mouse IgG (H + L)	1:1000	A21202	ThermoFisher Scientific, Waltham, MA, USA
Alexa Fluor 546	Donkey anti-rabbit IgG (H + L)		A10040	
	Goat anti- guinea pig IgG (H + L)		A11074	
	Goat anti-rat IgG (H + L)		A11081	

## Data Availability

The original contributions presented in the study are included in the article. Further inquiries can be directed to the corresponding authors.
